# Left retroperitoneal abscess caused by injury of the right knee

**DOI:** 10.1002/ccr3.2677

**Published:** 2020-01-28

**Authors:** Chie Imai, Hisanori Fujino, Anryu Nomura, Shinichi Sumimoto

**Affiliations:** ^1^ Department of Pediatrics Osaka Red Cross Hospital Osaka Japan

**Keywords:** antibiotics, methicillin‐susceptible *Staphylococcus aureus*, percutaneous drainage, retroperitoneal abscess

## Abstract

Minor trauma rarely triggers a retroperitoneal abscess. The patient' history, careful evaluation of their symptoms, and general examination with a detailed inspection of the skin are helpful to diagnose retroperitoneal abscess.

## Graphical abstract

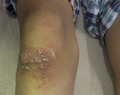

A 13‐year‐old Japanese boy was admitted to our hospital with remittent fever and left coxalgia following 4 days of antibiotic treatment without improvement. Upon admission, the patient appeared pale and complained of remittent fever, left buttock pain, and limping for 14 days. His past medical history was none. His temperature was 37.6°C, and other vital signs were normal. A physical examination revealed tenderness located in the left buttock. The patient could not extend the left hip joint and could not walk because of severe pain. He had injured his right knee 3 weeks before admission because of fall while playing soccer. (Figure 1) Laboratory results showed a white blood cell count of 26 340/μl with 84% neutrophils, C‐reactive protein of 10.0 mg/dl, and an erythrocyte sedimentation rate of 83 mm/h. Magnetic resonance imaging (MRI) showed a large 6 cm in diameter, retroperitoneal mass adjacent to the left iliopsoas muscle (Figure 2). Percutaneous drainage of a large amount of pus from the abscess was performed by placement of a pigtail catheter under ultrasonography guidance. The pigtail was removed after 10 days of intravenous antibiotics and monitoring the decrease in abscess size. Blood cultures of samples obtained at admission were negative. Cultures of pus from the retroperitoneal drainage and the wound of the right knee grew methicillin‐susceptible *Staphylococcus aureus*. The patient responded well to antibiotic treatment. Intravenous antibiotics, such as tazobactam/piperacillin, meropenem, and cefazolin, were given for total 5 weeks, followed by oral antibiotic, levofloxacin, for 5 days. The abscess in the retroperitoneal space had nearly disappeared after treatment. The wound of the right knee also healed.

**Figure 1:**
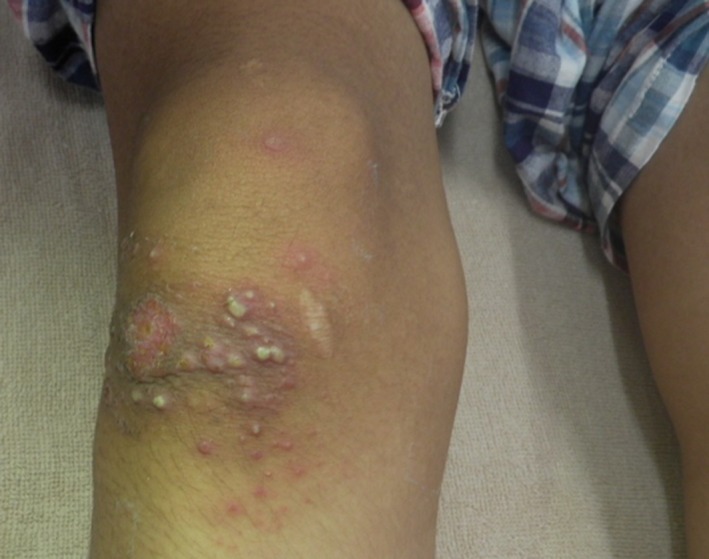
White blisters on the patient's right knee

**Figure 2:**
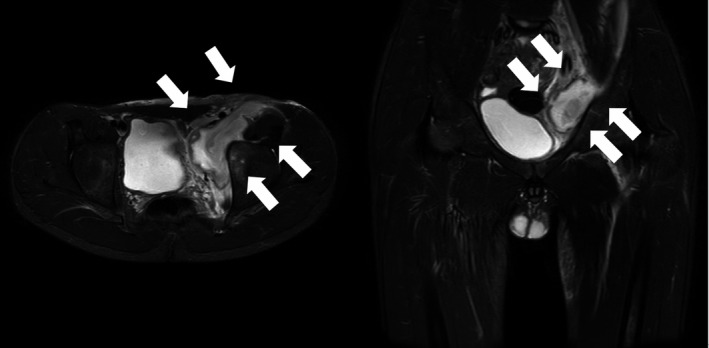
Axial and coronal T2 short tau inversion recovery sequence MRI‐revealed enhancement of retroperitoneal abscess adjacent to the left iliopsoas muscle

Retroperitoneal abscesses generally present with a high fever, lower abdominal pain, loin pain, thigh pain, and diarrhea.^1^ Retroperitoneal abscesses often result from gastrointestinal tract conditions like diverticulitis, appendicitis, or pancreatitis, or are related to previous surgery, remote infection, immunodeficiency, diabetes, or steroid intake.^2^, ^3^ Open cutaneous lesions rarely trigger a retroperitoneal abscess.^4^ Accurate diagnosis of retroperitoneal abscess is often delayed in pediatric patients because it is relatively uncommon in that population and the symptoms are nonspecific and nonlocalizing.^2^ The patient history, careful evaluation of their symptoms, and general examination with a detailed inspection of the skin are all helpful. If children with an open skin lesion have fever, are limping, and complained of buttock pain, retroperitoneal abscess should be included in the differential diagnosis. If retroperitoneal abscess is suspected, imaging such as ultrasonography, computed tomography (CT), and MRI should be performed quickly. A complete history including recent injuries and a high index of suspicion for the diagnosis are essential.

## CONFLICT OF INTEREST

None declared.

## AUTHOR CONTRIBUTIONS

CI: wrote the manuscript. HF: revised the manuscript. AN: revised the manuscript. SS: supervised the final draft.
